# A Smart System for the Contactless Measurement of Energy Expenditure

**DOI:** 10.3390/s22041355

**Published:** 2022-02-10

**Authors:** Mark Sprowls, Shaun Victor, Sabrina Jimena Mora, Oscar Osorio, Gabriel Pyznar, Hugo Destaillats, Courtney Wheatley-Guy, Bruce Johnson, Doina Kulick, Erica Forzani

**Affiliations:** 1 School of Engineering for Matter, Transport and Energy, Arizona State University, Tempe, AZ 85281, USA; mark.sprowls@asu.edu (M.S.); svictor4@asu.edu (S.V.); 2 Center for Bioelectronics and Biosensors, Biodesign Institute, Arizona State University, Tempe, AZ 85281, USA; smora2@asu.edu (S.J.M.); oosoriop@asu.edu (O.O.); gpyznar@gmail.com (G.P.); 3 Indoor Environment Group, Lawrence Berkeley National Laboratory, Berkeley, CA 94720, USA; hdestaillats@lbl.gov; 4 Mayo Clinic, Scottsdale, AZ 85289, USA; wheatley.courtney@mayo.edu (C.W.-G.); johnson.bruce@mayo.edu (B.J.); kulick.doina@mayo.edu (D.K.)

**Keywords:** smart home, point of care, metabolic rate, ambient biometrics, Internet of Things (IoT), digital medicine

## Abstract

Energy Expenditure (EE) (kcal/day), a key element to guide obesity treatment, is measured from CO_2_ production, VCO_2_ (mL/min), and/or O_2_ consumption, VO_2_ (mL/min). Current technologies are limited due to the requirement of wearable facial accessories. A novel system, the Smart Pad, which measures EE via VCO_2_ from a room’s ambient CO_2_ concentration transients was evaluated. Resting EE (REE) and exercise VCO_2_ measurements were recorded using Smart Pad and a reference instrument to study measurement duration’s influence on accuracy. The Smart Pad displayed 90% accuracy (±1 SD) for 14–19 min of REE measurement and for 4.8–7.0 min of exercise, using known room’s air exchange rate. Additionally, the Smart Pad was validated measuring subjects with a wide range of body mass indexes (BMI = 18.8 to 31.4 kg/m^2^), successfully validating the system accuracy across REE’s measures of ~1200 to ~3000 kcal/day. Furthermore, high correlation between subjects’ VCO_2_ and λ for CO_2_ accumulation was observed (*p* < 0.00001, R = 0.785) in a 14.0 m^3^ sized room. This finding led to development of a new model for REE measurement from ambient CO_2_ without λ calibration using a reference instrument. The model correlated in nearly 100% agreement with reference instrument measures (*y* = 1.06*x*, R = 0.937) using an independent dataset (N = 56).

## 1. Introduction

Obesity is an epidemic affecting 39.8% of the United States adult population as per latest data [1]. With the decreasing life expectancy by an estimated 5–20 years as a result of obesity-associated co-morbidities [2,3,4], obesity is clearly a major burden on society. The primary clinical intervention in obesity treatment is referred to as a deficit diet [5], where a medical professional outlines a dietary plan with the objective of reducing daily caloric intake from food to less than the total daily energy expenditure (TEE) of that person. Energy expenditure (EE) is the rate of energy consumption of a person at given time [6], with resting energy expenditure (REE) being a common conceptual form of EE [7] with great focus in obesity treatment. REE can be measured accurately using a principle referred to as indirect calorimetry [8,9], a technique based on the measurement of rates of O_2_ consumption and CO_2_ production by the well-established Weir formula [10]. Indirect calorimetry is used as the basis for all current U.S. Food and Drug Administration (FDA)-cleared/approved medical devices for REE measurement. These devices are widely available, yet measurement of EE is not commonly adopted by clinical practices. Instead, predictive equations for REE calculation are common practice, which is a potential cause for limited success in treatment of clinical obesity. These predictive equations have low accuracy [11]. For instance, for a given individual, REE differences of 900 kcal/day can be found between the calculated REE from Mifflin St. Jeor equation [12] and measured REE [13]. Many studies have observed similar findings [14,15,16,17]. Besides application to obesity medicine, personalized metabolic assessment has invigorated a passion for personal health monitoring and “calorie tracking” across the world, evidenced by worldwide Smartwatch adoption, although this technology has notably low accuracy [18]. As such, a convenient and accurate EE measurement technology could substantially benefit both obesity patients and individuals interested in their own personal health and physical performance.

Given known inaccuracies of predictive equations for REE, it is reasonable to question why EE measurement tools are not used extensively in clinical practice. An argument for this could be that the EE measurement using current technologies is a technique affected by widely recognized issues [19] that are a result of a fundamental design flaw of these devices: they typically require a subject to breathe into a mouthpiece, mask, or some object worn on the subject’s face. Wearing a facial accessory results in errors, including elevated VCO_2_ and VO_2_ from hyperventilation while wearing an object for breath gas collection on the face, or, alternatively, mistakenly breathing into an EE measurement medical device that is not fitted in an airtight manner to the subject’s face. These tools have the added operational issues related to wearing an object on the subject’s face, including discouraged repeated measurement due to discomfort, which is important given high day-to-day variability in REE, 11.8% [20]. Other considerations are related to sterilization of facial accessories, an aspect which increases both device cost (purchase of additional single-use accessories) and procedural time requirements (sterilization of facial accessories), and inherently increases the risk of transmission of various pathogens. In addition to usability concerns, many current FDA-cleared/approved technologies for REE measurement have reproducibility errors worse than ±10% (68% CI) by multiple definitions of reproducibility [21]. Alternatively, there is a technique for EE measurement from ambient sensors, in what is referred to as an “indirect calorimeter room” [22,23,24]. Current methodologies for development of an indirect calorimeter room are remiss in the sense that they are not portable and require extensive installation time; they are therefore unsuitable for widespread clinical adoption. There are only seventeen of these calorimeter rooms in the entire U.S., and an additional twenty-five in the rest of the world [24].

In this work, we present a novel EE measurement technique offering clear advantages over modern indirect calorimeters. The system presented here, the Smart Pad, resolves many of these issues, since the Smart Pad is not wearable and therefore fundamentally reduces analytical accuracy errors from wearable facial accessories and eliminates the operational issues mentioned above. In comparison with indirect calorimetry rooms, the Smart Pad, utilizing a mathematical model for VCO_2_ from a single sensor in the bulk of the measurement environment [25], can be transported from room to room. The Smart Pad’s room conditioning involves the connection of the inlet and/or outlet ventilation fan/s to the Smart Pad actuator (see details below). A previous work [26] reports on the analytical accuracy of the Smart Pad system in one simple configuration within a clinical setting and performing measurements on 20 subjects using a reference instrument for EE measurement in parallel with the Smart Pad. This manuscript builds upon that work by optimizing the operating parameter of EE measurement resolution while also maximizing measurement accuracy. Eventually, the system could be implemented synergistically with other recent advancements in the field of medical device engineering, where there is a strong trend towards non-invasive [27,28,29,30] or totally contactless [31,32,33,34,35,36,37] measurement.

The assessment of a room’s air exchange rate, referred to as “λ” in this work, is a critical challenge for measurement of REE from ambient CO_2_ accumulation patterns. λ is a common consideration in hospitals striving to minimize pathogen transmission [38], schools [39] where ventilation can influence learning performance [40], and office buildings [41] to prevent incidences of sick building syndrome [42]. It can be approximated via quantitative assessment of CO_2_ concentration decay in environments with no CO_2_ sources (i.e., no occupants), and a well-established CO_2_ model has been studied extensively [43,44,45]. Further, the CO_2_ decay modelling procedure has shown good agreement with reference methods that use a tracer gas [46,47], suggesting that the technique is accurate in unoccupied environments. Recently, λ values obtained from decay modelling have been applied to CO_2_ accumulation data for determination of human CO_2_ production rate (VCO_2_) in cognitively stressed subjects [48]. Our previous study [26] accounted for λ by calibration with a reference instrument’s VCO_2_ measurements during CO_2_ accumulation in an 8 m^3^ sized room and the technique was shown to be accurate for assessment of an individual’s VCO_2_. Here, we implemented this EE assessment technique by utilizing a fully developed and automated Smart Pad system while investigating two methods of assessing λ values: Method A, named “λ_Acc_”, with λ values assessed from CO_2_ accumulation data collected in presence of a subject of known VCO_2_; and Method B, named “λ_o_”, with λ values assessed from CO_2_ decay data collected in the absence of the subject. Both methods were carried out in a medium-sized test room. Additionally, a strong correlation between λ_Acc_ and VCO_2_ was observed, which led to the development of a novel model for VCO_2_ and REE assessment within an occupied room that does not rely directly on λ calibration using a reference instrument [26] or λ assessment from CO_2_ decay rate [46]. The model shows good agreement for REE assessment when compared to independent measurements from a reference instrument.

## 2. Materials and Methods

### 2.1. Smart Pad System Operating Equations and Measurement Technique

The following equations were used, derived in a previous work [25]. Equation (1) describes a physical environment where CO_2_ (concentration units expressed in part-per-million, ppm) accumulates due to a human’s occupancy; air exchange to the surrounding environment occurs and is characterized by λ (h^−1^), specifically referred to as λ_Acc_ when it is applied to CO_2_ accumulation data. k_gen_ is the CO_2_ generation rate in the environment (ppm h^−1^) due to occupant CO_2_ production from breath:

(1) $$[CO_{2}]={\left[CO_{2}\right]}_{0}+\frac{k_{gen}}{\lambda_{ACC}}\;\left(1-e^{-\lambda_{ACC}t}\right)+{\left([CO_{2}\right]}_{i}-{\left[CO_{2}\right]}_{0})e^{-\lambda_{ACC}t}$$

Previous findings [26] suggested that k_gen_ was correlated to the actual k_gen_′ via an environmental correction factor (CF_env_) of 1.143, an empirical constant determined to correct for mean error bias originating from any source not accounted for in the “ideal model” used in Equation (1), e.g., imperfect mixing, CO_2_ sensor response time, etc., and is used in the model as follows:

(2) $$k_{gen}^{\prime }=k_{gen}CF_{env}$$

Once λ_Acc_ is estimated, k_gen_ is then assessed for the subject and used to calculate k_gen_′ from Equation (2). k_gen_’ is then used in the following equation to determine the value of VCO_2_ (mL/min):

(3) $$VCO_{2}={k_{gen}}^{\prime }*V_{Room}*CF_{STPD}/60$$

where VCO_2_ is the subject’s volumetric production of CO_2_ (mL/min), V_Room_ is the room’s volume (mL), and CF_STPD_ (dimensionless) is a correction factor used to convert measured ambient VCO_2_ to standard temperature, pressure, and dry conditions (STPD). The correction factor was calculated as follows:

(4) $$CF_{STPD}=\frac{P_{bar}-P_{H20}}{760}*\frac{273}{T+273}$$

where P_bar_ (mmHg) is barometric pressure, P_H20_ is partial pressure of H_2_O (mmHg), and T represents temperature within the environment (°C). EE (kcal/day) of the subject was calculated using a simplified version of the Weir formula [10] (Equation (12) of the original publication) that assumes a constant respiratory quotient (RQ) of 0.85 (see more details in Supplementary Materials, Section S1):

(5) $$EE\;\left(\mathrm{kcal}/\mathrm{day}\right)=3.941*\frac{VCO_{2}}{RQ}+1.106*VCO_{2}$$

(6) $$RQ=\frac{VCO_{2}}{VO_{2}}$$

In addition, the λ values assessed from CO_2_ decay data collected in absence of the subject are referred to as λ_0_ (hour^−1^) and were estimated from a standard first-order decay equation as follows:

(7) $$[CO_{2}]={\left[CO_{2}\right]}_{0}+\;{\left([CO_{2}\right]}_{i}-{\left[CO_{2}\right]}_{0})e^{-\lambda_{0}t}$$

where λ_o_ is the air exchange rate (hour^−1^) in the absence of a subject, and distinctively different from λ_Acc,_ the air exchange rate (hour^−1^) in the presence of a subject (Equation (1)).

### 2.2. Smart Pad: Physical Characteristics, Design, and Testing Protocol

**Figure 1:**
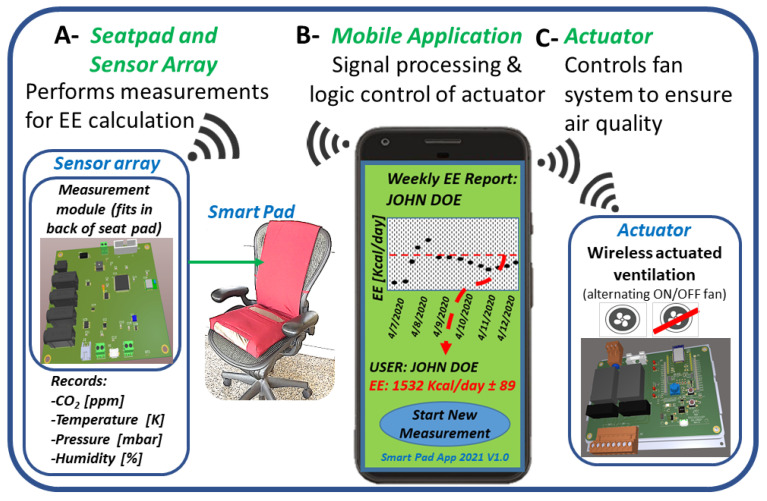
Scheme showing the three wireless connected components of the Smart Pad system: (**A**): Sensing module; (**B**): Mobile application (presented user interface is not yet fully developed); (**C**): Actuator system.

**Figure 2:**
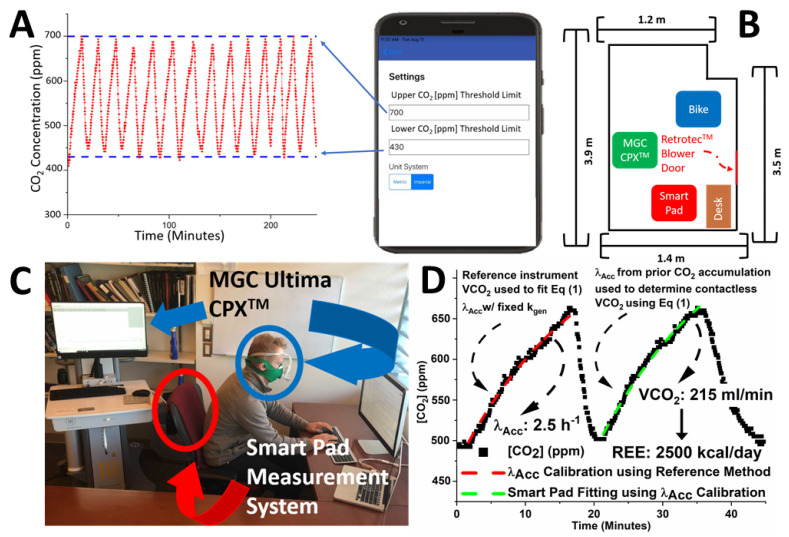
(**A**) Graphic showing Smart Pad mobile application and settings allowing for precise control of CO_2_ concentration. (**B**) Graphic showing top view of room layout and locations of various objects. (**C**) Subject during test procedure for parallel REE measurement with Smart Pad and reference instrument. (**D**) Graphical demonstration of Smart Pad’s k_gen_ assessment with two sets of CO_2_ accumulation data, using known λ_Acc_ from first set of CO_2_ accumulation data. In the first CO_2_ accumulation cycle, the CO_2_ accumulation fitting is performed using Equation (1) and the known k_gen_ from the reference instrument. In the second CO_2_ accumulation cycle, λ_Acc_ from the first accumulation cycle is used to determine k_gen_ using Equation (1). K_gen_ is then used to calculate EE via a combination of Equations (3)–(6).

The Smart Pad system is comprised of three components. (A) The measurement system, a wireless device with an embedded sensor array located in the rear of a seat pad (Figure 1A). This array includes sensors for carbon dioxide (CO_2_) and environmental conditions (humidity, temperature, and barometric pressure) necessary for REE measurement from Equations (1)–(7). (B) The actuator system, which controls the room’s ventilation (Figure 1C) using logic defined by user-selected maximum and minimum threshold levels set for CO_2_ using an iPhone (iOS) application (see Figure 2A). (C) The mobile (iOS) application, which hosts algorithms with defined functions of sensing, actuation, information processing, and data analytics (Figure 1B). The mobile application is under development and currently has functional wireless and automated sensing and actuation abilities.

The measurement system’s CO_2_ measurements are based on non-dispersive infrared (NDIR) absorption by a commercial CO_2_ sensor (Telaire^TM^ 7001D CO_2_ monitor, Test Equipment Depot, Woburn, MA, USA). A brand-new CO_2_ measurement device was used for the entire study and factory calibration was validated using a CO_2_ reference gas and outdoor air, which is well known to contain approximately 415 ppm CO_2_ in most areas of the world [49], to the rated accuracy of the CO_2_ measurement device (±50 ppm). An inlet tube with a diameter of approximately 0.3 cm was connected to the sample inlet port of the CO_2_ sensor, sealed with parafilm, and then allowed to rest approximately 5 cm out of the seatpad. The CO_2_ sensor was connected to the sensing module, which also includes embedded temperature, barometric pressure, and relative humidity sensors. The iOS application was programmed to collect data with a resolution of 1 measurement/5 s for all measurements. It is worth noting that the Smart Pad CO_2_ sensor does not collect real-time respiration CO_2_ patterns. The sensor is meant to collect changes in environment CO_2_, which are reflective of the metabolic rate of the individual present in the environment.

The Smart Pad was installed on a chair in a rectangular room with a measured volume of 14.0 m^3^, which accounts for the size of objects, and approximate dimensions of 2.7 m × 1.4 m × 3.7 m (see layout and object locations in Figure 2B). The room was well sealed using tape to cover interstitial space between ceiling tiles and cardboard panels to block the room’s built-in HVAC (heating, ventilation, and air conditioning) system. A special Retrotec^TM^ (https://retrotec.com/, accessed 23 December 2021) blower door was installed on the doorway fixed with an outlet fan and also a transparent plastic window allowing for observation of test subjects. The λ_o_ values for this room ranged from 1.5 to 3 h^−1^, which is in agreement with similar findings for administrative offices [50], suggesting that significant air exchange will still occur even with the room mostly sealed from the surrounding environment (Retrotec^TM^ door’s opening for an outlet fan was likely the primary air exchange medium). Additionally, two small (approximately 40 cm diameter) mixing fans were pointed towards each other at opposite corners of the measurement environment to support mixing of CO_2_ in the room. The fans were left on continuously during both CO_2_ accumulation and decay periods. Supplementary Materials, Section S2 provides more details on system design, standard operation, and testing environment.

The system prevents CO_2_ exposure to levels above a maximum threshold CO_2_ through ventilation actuation, given that exposure to a high concentration of CO_2_ can compromise a person’s cognitive functions [51,52]. Figure 2A illustrates the concept: when the CO_2_ levels reach a level of 700 ppm, the system actuates the ventilation, which causes the CO_2_ level to decrease. Once the minimum threshold CO_2_ is reached, the SmartPad turns off the room air ventilation. This enables the accumulation of CO_2_ in the room and causes significant changes in CO_2_ in the room environment, which allows for the assessment of VCO_2_ through data analysis. This cycle repeats until the subject departs from the environment and many sequential CO_2_ accumulation cycles are often recorded. This design makes the system uniquely innovative: collecting CO_2_ data with each cycle, which can be analyzed and provides access to multiple EE data points for high-temporal resolution monitoring while also preventing exposure to unhealthy CO_2_.

Experiments were performed using three distinct methodologies. (1) Sequential REE assessments (see Section 2.3 and Section 2.5): subject remains seated in room while REE measurement cycles are recorded every 10–30 min. (2) Sequential Exercise VCO_2_ assessments (see Section 2.4): subject remains in room while exercise VCO_2_ measurements are recorded every 5–10 min. (3) Sequential CO_2_ accumulation and decay assessments (see Section 2.6): subject remains seated in room for a singular REE assessment and then immediately departs upon CO_2_ accumulation to a particular threshold limit.

### 2.3. CO_2_ Measurements for REE Assessment

Subject 1 performed the first phase of this research work. The physical characteristics of the subject are shown in Table 1. Subject 1 performed a total of 113 measurements with both systems: Smart Pad and a reference instrument for VCO_2_ and REE measurement, the MGC Ultima CPX^TM^ Indirect Calorimeter, an FDA 510(k) cleared system [53], considered one of the best breath-by-breath indirect calorimeters. The basic configuration of the experiment is shown in Figure 2C, with Subject 1 performing parallel reference instrument and Smart Pad measures by wearing the MGC Ultima CPX^TM^ mask (connected to the metabolic cart) while occupying a room with the Smart Pad (sensing module) installed on a chair. The MGC Ultima CPX^TM^ system measures both VCO_2_ and VO_2_ by direct physical measurement of flow rate (via pitot tube), O_2_ concentration (via galvanic cell potential), and CO_2_ concentration (via NDIR absorption) [53]. The 113 REE measurements were split between 5 CO_2_ threshold ranges of 500–600 ppm, 500–625 ppm, 500–650 ppm, 500–675 ppm, and 500–700 ppm. Multiple CO_2_ accumulation cycles were measured sequentially. Λ_Acc_ was assessed by recording a first CO_2_ accumulation curve and fitting the data with reference k_gen_ value assessed from the reference instrument’s VCO_2_ as shown in Figure 2D. In parallel, the subject’s VCO_2_ measures were assessed from the Smart Pad system (shown in Figure 1 and Figure 2C). The subject fasted for 8 h prior to each REE assessment to minimize the well-studied thermic effect of food, which is known to increase EE by a significant margin [54]. The subject remained seated at a desk while measurement cycles of CO_2_ accumulation data were recorded for REE assessment. See Supplementary Materials, Section S3 for more details on the experimental protocol.

### 2.4. CO_2_ Measurements for Exercise Energy Expenditure Assessment

Subject 1 performed N = 46 simultaneous Smart Pad and MGC Ultima CPX^TM^ exercise VCO_2_ assessments using a stationary exercise bike. The subject often biked at a high intensity; however, no formal measure of exertion was recorded. The bike was facing the Smart Pad unit and was approximately 1 m away from the seat pad (see Figure 2B). See Supplementary Materials, Section S4 for more details on the experimental protocol.

### 2.5. CO_2_ Measurements in Subjects of Varying Body Mass Index and REE

Test subjects were recruited with approval from Arizona State University’s institutional review board (STUDY00006547) after giving consent to participate in the study and were asked to perform six sequential MGC Ultima CPX^TM^ and Smart Pad measurements at the CO_2_ threshold range of 500–650 ppm (one for λ_Acc_ assessment from reference instrument VCO_2_ and five comparative measurements). This was done to determine whether subject BMI, VCO_2_, or EE had any significant effect on Smart Pad accuracy or λ_Acc_. The physical characteristics of each subject are shown in Supplementary Materials (Table S1).

### 2.6. Evaluative Study of Room Air Exchange Rate (λ_Acc_ vs. λ_o_)

In the scientific literature [43,44,45,48], there is a general assumption that λ_0_ from CO_2_ decay following subject departure is in agreement with λ_Acc_. In this study, a reference instrument for VCO_2_ measurement was used to assess λ_Acc_ with high accuracy, as shown in Figure 2D, and λ_Acc_ was compared with λ_0_ assessed from CO_2_ decay data, collected following subject departure from the room. A total of twenty-six (N = 26) CO_2_ accumulation periods and subsequent CO_2_ decay periods were collected with the actuator system turned off. A minimum of five (N = 5) assessments were performed for each of the following CO_2_ concentration ranges (ppm): 500–600, 500–625, 500–650, 500–675, and 500–700. For the 500–650 ppm data set, CO_2_ accumulation and subsequent CO_2_ decay was collected using N = 1 measurement from each subject #2–5 and N = 2 measurements from Subject 1.

### 2.7. Data Analysis

Fitting of CO_2_ accumulation data or CO_2_ decay data was performed with Equation (1) and Equation (7), respectively, using the least square error method. In general, errors between the Smart Pad and reference method were assessed as: Error (%) = [(REE_Smart Pad_ – REE_MGC CPX_)_/_REE_MGC CPX_] × 100%. For biking assessments, VCO_2_ was used to calculate errors in place of REE given the reasoning discussed below.

## 3. Results and Discussion

### 3.1. Optimization of CO_2_ Measurement Range for REE Assessment—Single Subject Study

**Figure 3:**
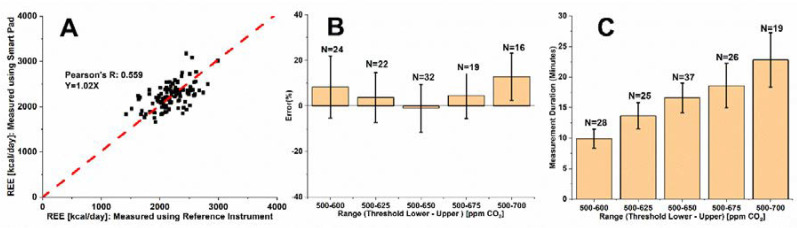
(**A**) Correlation of all N = 113 Smart Pad REE measures with reference instrument measures across all CO_2_ threshold ranges; (**B**) Effect of upper and lower threshold range settings on Smart Pad accuracy for REE measurement; (**C**) Effect of threshold range settings on measurement duration.

To study the effect of the Smart Pad’s upper and lower CO_2_ threshold range settings on REE measurement accuracy, a single subject performed a total of 113 measurements as follows: 24 measurements for a range between 500–600 ppm, 22 measurements for a range between 500–625 ppm, 32 measurements for a range between 500–650 ppm, 19 measurements for a range between 500–675 ppm, and 16 measurements for a range between 500–700 ppm. Comparative measurements between the REE values obtained from Smart Pad and the reference instrument are summarized in Figure 3.

Figure 3A shows relatively good correlation (*y* = 1.02*x*) between parallel REE measurements recorded by the Smart Pad and the reference instrument, with an averaged mean bias of 3.3% across all measurement conditions suggesting that Equation (1) is robust overall across all five CO_2_ threshold ranges examined within this work. However, the relatively low Pearson’s R of 0.559 indicates that further optimization of the measurement error is required. Figure 3B details the effect of Smart Pad threshold range on system precision and accuracy, showing clear differences in measurement characteristics across differing threshold ranges. The 500–650 ppm CO_2_ concentration range was concluded to be the most accurate and precise operating range, with performance characteristics of −1.0% ± 10.5% (SD) for the subject across N = 32 total REE measurements. Figure 3C shows the effect of differing CO_2_ threshold ranges on measurement duration. Clearly, measurement duration increased consistently with increasing CO_2_ threshold range. From Figure 3B,C, one may reasonably conclude that the 500–650 ppm CO_2_ threshold range provides optimal precision and accuracy, with REE measurement accuracy of −1.0% ± 10.5% for a 14–19 min measurement duration. Repeated measures can improve the Smart Pad accuracy significantly (see Supplementary Materials, Section S7). In addition, the Smart Pad’s maximum error after λ_Acc_ calibration using a reference instrument (11.5% for ±1 SD) is comparable to errors reported for well-respected FDA-cleared/approved medical devices for REE measurement [21]; for example: Korr ReeVue^TM^ (11.1% for ±1 SD) and MGC CPX Ultima^TM^ (12.2% error for ±1 SD).

### 3.2. Optimization of CO_2_ Measurement Range for Exercise Energy Expenditure Assessment—Single Subject Study

**Figure 4:**
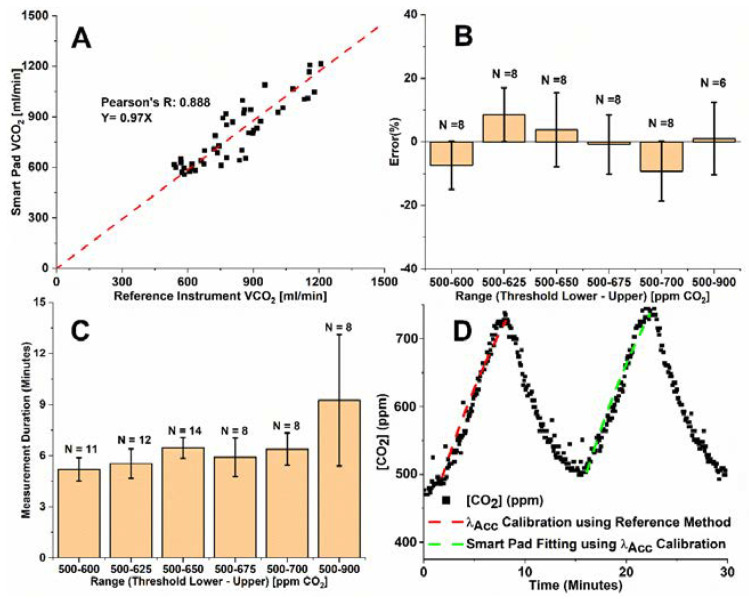
(**A**) Correlation of all N = 46 Smart Pad exercise VCO_2_ measures with reference instrument measures across all CO_2_ threshold ranges; (**B**) Effect of upper and lower threshold range settings on Smart Pad accuracy for exercise VCO_2_ measurement; (**C**) Effect of threshold range settings on the measurement duration for a biking subject. (**D**) Sample data analysis for 500–675 ppm threshold range.

Figure 4A–C shows Smart Pad VCO_2_ measurement performance for exercise (biking on a fixed bike) in a similar analysis format presented in Figure 3 for REE data.

Figure 4A shows high correlation between parallel exercise VCO_2_ measurements from the Smart Pad and reference instrument, with an average mean bias of −0.8% across all measurement conditions, suggesting that Equation (1) is robust across all measurement conditions. Figure 4B shows how the Smart Pad app’s CO_2_ threshold range affects accuracy for VCO_2_ measurement, with the 500–675 ppm threshold range showing optimal measurement with errors of −0.8% ± 9.3% (SD). Further, Figure 4C shows the effect of CO_2_ threshold range on measurement duration. A less pronounced increase in measurement duration with increasing size of CO_2_ threshold range was observed compared to the set of experiments for REE. One possible explanation is that the effort level on the bike was not a control variable of the study. Measurement duration for the lowest error range (500–675 ppm) corresponded to only 4.8–7 min of contactless VCO_2_ measurement. EE calculation via the Weir formula in Equation (5) assumes an RQ value of 0.85. However, the RQ value may vary significantly during the course of exercise [55,56], and may potentially be a source of error for Smart Pad under exercising conditions. For that reason, exercise EE accuracy is not reported.

### 3.3. Smart Pad REE Assessment–Multiple Subject Study

Five subjects were studied across a wide range of BMIs and REEs using the reference instrument and Smart Pad at the optimized CO_2_ threshold range of 500–650 ppm. The procedure shown in Figure 2D was followed, which uses a reference instrument to calibrate for λ_Acc_. Each subject performed a minimum of five independent measurements, and a total of 52 REE measurements were performed on all the subjects. Figure 4A–C summarizes the effects of BMI on measurement accuracy, the effects of subject REE on measurement accuracy, and the effects of subject REE on Smart Pad measurement duration.

**Figure 5:**
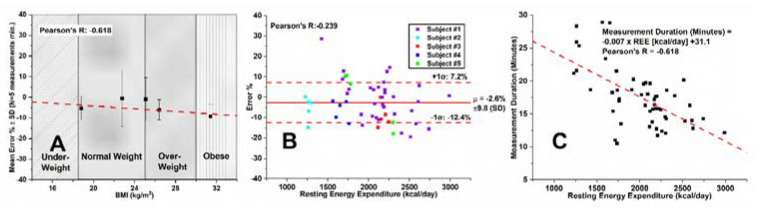
Smart Pad system performance for REE assessment at optimal CO_2_ range in 5 subjects: (**A**) Smart Pad mean error % ± SD for REE measurement categorically grouped using BMI and ranging from just above the underweight cutoff (18.8 kg/m^2^) to obese (31.4 kg/m^2^); (**B**) Bland–Altman plot for REE measurement accuracy across various subject REEs; (**C**) Effect of subject REE on Smart Pad measurement duration at 500–650 ppm CO_2_ threshold range.

Figure 5A shows Smart Pad’s REE measurement mean error as a function of BMI. Mean error biases across all five subjects were within ±10%, an analytically accepted error for FDA-cleared devices in the U.S. [21]. Figure 5A’s slope was determined to be statistically insignificant (*p* = 0.267), suggesting that the Smart Pad is accurate over a wide range of BMI values. In addition, Figure 5B shows a Bland–Altman plot of Smart Pad error % for REE measurement, and confirms low mean bias (µ = −2.6%) between the MGC Ultima CPX^TM^ and Smart Pad and an insignificant (*p* = 0.088) effect of REE on system accuracy across all N = 52 measurements performed on the N = 5 subjects. These findings suggest that the Smart Pad is accurate for a wide range of metabolic rates and body types.

Figure 5C shows a plot between subject REE and Smart Pad’s measurement duration. The general trend in the plot indicates decreasing measurement duration with increasing REE. This is an intuitive result based on system design, as, while holding upper CO_2_ level constant, elevated CO_2_ generation rates will reach the upper CO_2_ level more quickly than would a reduced CO_2_ generation rate. In general, from regressions shown in Figure 5A,B, we conclude that neither subject BMI nor subject REE have statistically significant effects on Smart Pad accuracy when using a reference instrument to calibrate for λ_Acc_.

### 3.4. Smart Pad REE Assessment—New Smart Pad System Model

#### 3.4.1. Evaluation of Air Exchange Rate Assessment from Decay Model (λ_o_) vs. Accumulation Model (λ_Acc_)

A room’s air exchange rate (λ) can be determined safely within indoor environments with the estimation of subject VCO_2_ during CO_2_ accumulation periods (λ_Acc_) [43,57], or recording CO_2_ decay following exit of human occupants from an environment (λ_o_) [43,44,45,48]. Previous reports of λ assessed from CO_2_ accumulation data (λ_Acc_) [43,57] were based on predictive equations for REE (e.g., [12,58]). However, it is widely reported that assessment of REE based on REE predictive equations is often inaccurate [11,13]. In this work we focus on assessing λ_Acc_ with high accuracy so that we can formally study the correlation of λ_Acc_ vs. λ_o_ and λ_Acc_ vs. VCO_2_ (k_gen_). In order to do this, we build upon the previous work [26] in which λ_Acc_ was assessed using a reference instrument. Here, we build upon that work by using a reference instrument to provide a high-accuracy value of λ_Acc_ (using k_gen_ derived from an FDA 510(k)-cleared device’s VCO_2_ measure) and λ_0_ from CO_2_ decay in the exact same environment following the subject’s departure.

**Figure 6:**
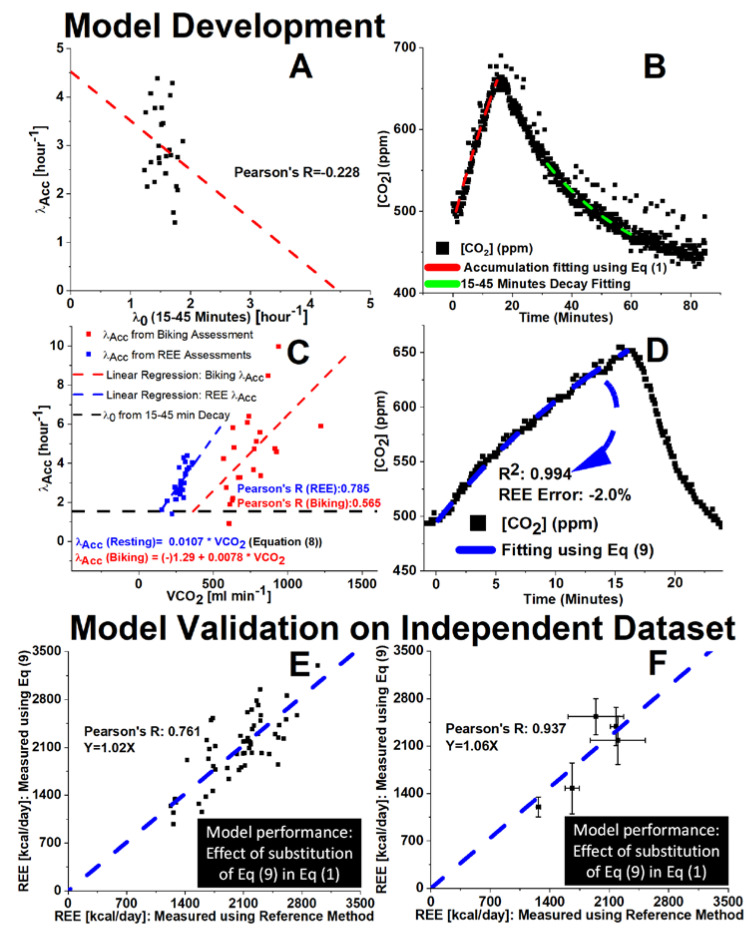
Results of λ_Acc_ correlative study. (**A**) λ_Acc_ vs. λ_0_ scatter plot showing little to no correlation (R = −0.228) for N = 26 sequential CO_2_ accumulation/decay analyses; (**B**) Sample data analysis for sequential CO_2_ decay/accumulation experiments, (**C**) Correlation between VCO_2_ measured from the MGC Ultima CPX^TM^ and λ_Acc_ as assessed from Equation (1) with Smart Pad system; (**D**) Sample data analysis using Equation (9), € Correlation between REE measured using Equation (9) and REE measured using MGC Ultima CPX^TM^ for N = 56 total measurements on N = 5 subjects; (**E**) Correlation for the same dataset € (**F**) but presenting mean REE ± SD for each subject’s measurements.

It is worthy to mention that in general, there is an assumption within the scientific community that these parameters (i.e., λ_Acc_ and λ_0_) are in agreement. Figure 6A shows the results of λ_Acc_ vs. λ_0_ from this study. Λ_Acc_ values were obtained with a procedure described in Figure 2D and λ_0_ values were assessed from decay data as shown in Figure 6B, right branch of CO_2_ data. The data shown in Figure 6A were obtained from Subjects 1–5’s data for a total of N = 26 measurements, including sequential CO_2_ accumulation and decay at the five threshold ranges (500–600, 500–625, 500–650, 500–675, and 500–700 ppm) for a minimum of N = 5 sequential measurements for each CO_2_ threshold range. It can be observed that in our study there was no agreement between λ_Acc_ vs. λ_0_ (R = −0.228). Additionally, there was relatively low variance in λ_0_ assessed from CO_2_ decay, with sample means of 1.9 ± 0.2 h^−1^ (CV = 11%), 1.9 ± 0.2 h^−1^ (CV = 11%), and 1.8 ± 0.2 h^−1^ (CV = 11%) for 15, 30, and 45 min of CO_2_ decay, respectively.

Figure 6C shows λ_Acc_ assessed from Equation (1) using Smart Pad in comparison with VCO_2_ measured from the reference instrument. A high correlation can be observed between those parameters (λ_Acc_ and VCO_2_) for resting state (R = 0.785, *p* < 0.00001) and a moderate correlation for exercise state (R = 0.565, *p* < 0.01). A possible explanation for these correlations is that the λ_Acc_ is strongly influenced by changes in mass transport factor due to the subject’s presence in the room, leading to a rise in air exchange across openings connecting the room with adjacent spaces. Such changes may be due to increased convective transport associated with body movements and air density gradients due to human heat dissipation [59,60,61]. These effects are maximized during biking tests, where the subject’s heat flux is elevated from exercise and there is additional air convection due to pedaling.

#### 3.4.2. New Smart Pad System’s Model Validation

In order to accurately measure contactless EE in free-living conditions, it is important to assess k_gen_ from the CO_2_ concentration profiles described by Equation (1). To do so, it is essential to assess the environment’s air exchange rate during occupancy, λ_Acc_. Figure 6A demonstrates that λ_0_ is not an adequate predictor of λ_Acc_. Using the strong observed correlation between λ_Acc_ and VCO_2_ as shown in Figure 6C, this issue may be resolved by simultaneous assessment of λ_Acc_ and VCO_2_ without immediate prior calibration using a reference instrument. This is only possible given that VCO_2_ correlated so strongly with λ_Acc_ in the test environment. As such, λ_Acc_ can be ascertained via regression utilizing the relationship found in this work (Figure 6C), shown as follows:

(8) $$\lambda_{CO_{2}\;Acc}=\alpha *\;VCO_{2}\;$$

In this study, α = 0.0107 (h^−1^ min^−1^ mL), and one might expect the term to vary in differing measurement environments. By introducing Equation (8) in Equation (1), we obtain Equation (9) as follows:

(9) $$[CO_{2}]={\left[CO_{2}\right]}_{0}+\frac{1}{\beta }\;\left(1-e^{-\beta *k_{gen}*t}\right)+{\left([CO_{2}\right]}_{i}-{\left[CO_{2}\right]}_{0})e^{-\beta *k_{gen}*t}\;$$

where variable meanings and dimensions are the same as for Equation (1), except for a new term, β (ppm^−1^), which is as follows:

(10) $$\beta =\left(\frac{\alpha }{60}\right)*CF_{Env}*V_{Room}*CF_{STPD}$$

β considers unit conversion (mL min^−1^ to ppm h^−1^) and the factor from Equation (8) that represents “the change in λ_Acc_ resulting from a 1 unit increase in “k_gen_”.

An independent dataset of N = 56 measurements from N = 5 subjects was used to validate accuracy for the determination of energy expenditure from these new equations. This dataset contained no overlap in individual measurements with regards to the model development dataset. The results of the application of Equation (9) to independent data are shown in Figure 6E,F. Figure 6D shows an example of fitting Equation (9) to a CO_2_ accumulation profile for a subject of our study. Figure 6E shows the correlation plot of the Smart Pad’s REE measurement from Equation (9) with the REE measurement from the MGC Ultima CPX^TM^. The results show a low mean bias relative to the reference instrument: slope = 1.02 versus 1.00 for ideal measurement with R ~0.8. Taking the mean of multiple repeated REE measures (Figure 6F) increases correlation with the reference instrument and a Pearson’s R = 0.937, which is a logical consequence of standard error [62] and the general ability of measurement devices to mitigate imprecision using repeated measures. This new model represents an important step forward from the progress made in [26] towards development of a contactless IoT device for REE measurement, given that the promising application of Equation (9) requires no direct calibration for λ_Acc_ using a VCO_2_ measurement reference instrument. The data presented in Figure 6E is displayed in comparison with current FDA 510(k)-cleared (authorized for prescription use) technologies for metabolic assessment in Table 1 below.

**Table 1:** Comparison of Smart Pad Clinical Validation study (this work) to Current Technologies.

Indirect Calorimeter Medical Device (FDA Authorized for Prescription Use)	Single Breath Gas, VCO_2_ or VO_2_ (mL/min), Measurement Accuracy	Regulatory Considerations	Anatomical Contact Sites
Vyaire MasterScreen CPX^TM^	±50 mL/min (VCO_2_) [63]	510(k) Cleared [63]	Face
Vyaire Oxycon Pro^TM^	±50 mL/min (VCO_2_) [64]	510(k) Cleared [64]	Entire head
Vyaire Vyntus CPX^TM^	±50 mL/min (VCO_2_) [65]	510(k) Cleared [65]	Entire head + torso
Vyaire Oxycon Mobile^TM^	±50 mL/min (VCO_2_) [66]	510(k) Cleared [66]	Face
Microlife MedGem^TM^	Y = 0.83X (R = 0.81) (VO_2_) [67]	510(k) Cleared [67]	Mouth
MGC Ultima CPX^TM^ Indirect Calorimeter	±3% (exhalation rate)	[53,68] Cleared	Face
Smart Pad: 14–19 Minute Measurement	±45 mL/min (VCO_2_)	Meets 510(k) Standard	N/A (contactless)
Smart Pad: 14–19 Minute Measurement	y = 1.05x (R = 0.82) (VCO_2_)	Meets 510(k) Standard	N/A (contactless)

## 4. Conclusions

A new contactless system named Smart Pad was evaluated. The system displayed promising accuracy characteristics for contactless resting energy expenditure (REE) and exercise CO_2_ production rate (VCO_2_) measurements in a medium size room after calibrating for air exchange rate with a VCO_2_ measurement reference instrument method once. The Smart Pad is capable of performing accurate REE measurements in 14–19 min and exercise VCO_2_ measurements in 5–7 min after calibrating with the reference instrument once. In this configuration, measurement characteristics were comparable to multiple wearable (i.e., with facial accessories) FDA-cleared devices as reported by another study. Additionally, via nonlinear regression of sequential CO_2_ accumulation and decay data in the same environment using a high accuracy FDA-cleared medical device, observed differences in air exchange rate could be rationalized statistically in terms of a strong correlation with VCO_2_. This led to development of a new model for REE assessment from ambient CO_2_, which does not require a direct air exchange rate calibration using a reference instrument method or CO_2_ decay rate. The model shows good agreement for REE assessment (y = 1.06x, R = 0.937) when evaluated on a dataset independent from the one used to develop the model and measurements for the same subjects were averaged. The model has been demonstrated in a few subjects as a proof-of-concept, and it is currently being tested in a larger study. So far, the new model was concluded to meet the FDA 510(k) standard for authorization of a medical device for prescription use by comparison with five contact-based indirect calorimeters currently used today in clinical practice, with these comparisons shown in Table 1.

Future work will focus on validation of Equation (9) in a new environment and the development of a contactless physical fitness test.

## 5. Patent and Competing Interest Statement

Arizona State University owns intellectual property related to the measurement technology: US Patent Application No. 20210048206. The US Patent Application No. 20210048206 has been licensed to TF Health Co. Dr. Erica Forzani is co-founder of TF Health Corp., Tempe, AZ, USA.

## Data Availability

Some raw sample data presented in this study are available in Supplemental Information. For more inquiries concerning data, please contact the corresponding author.

## Supplementary Materials

The following supporting information can be downloaded at: https://www.mdpi.com/article/10.3390/s22041355/s1: S1. Smart Pad System Operating Equations and Measurement Technique; Figure S1: Sample regressions for Smart Pad system; S2. Smart Pad: Physical Characteristics, Design, and Testing Environment; Figure S2: Graphic illustrating Smart Pad assembly; Figure S3: Graphic demonstrating how the Retrotec^TM^ door was sealed for every experiment; Figure S4: The Smart Pad actuator system; Figure S5: The Smart Pad room’s carbon dioxide level distribution; S3. CO_2_ Accumulation Range Optimization for REE assessment; S4. CO_2_ Accumulation Range Optimization for Exercise assessment; S5. CO_2_ Accumulation Range Optimization for REE assessment: Results; S6. Additional information, including Table S1. Physical Characteristics and Body Type Classification, Figure S6: Sample raw data for many sequential REE assessments using a CO_2_ threshold range of 500–650; Figure S7: Sample baseline adjusted CO_2_ fitting; Table S2: Figure S7 Analysis Results; Figure S8: Sample fitting of Equation (9) on CO_2_ accumulation data; Table S3: Figure S8 Analysis Results; S7. Discussion: Compensation for Imprecise Measurements using Repeated Measures; Table S4: Figure S8 Analysis Results; Figure S9: Smart Pad accuracy using Equation (9) and extrapolating based on widely accepted implications of standard error. References [10,21,26,27,53,67,68,69,70,71,72] are cited in Supplementary Materials.
