# Supplementary material for: A Smart System for the Contactless Measurement of Energy Expenditure
Source: Sensors (Basel). 2022 Feb 10;22(4):1355. doi: 10.3390/s22041355 (PMC8963031; doi:10.3390/s22041355)
Supplement: Supplementary file 1 [file sensors-22-01355-s001.zip › Supplemental Information - A Smart System for the Contactless Measurement of Energy Expenditure.pdf]

Supplementary Materials

A Smart System for the Contactless Measurement of Energy Expenditure

Mark Sprowls [1,2], Shaun Victor [1,2], Sabrina Jimena Mora [2], Oscar Osorio [2], Gabriel Pyznar [2], Hugo Destaillats [3], Courtney Wheatley-Guy [4], Bruce Johnson [4], Doina Kulick [4] and Erica Forzani [1,2,*]

[1] School of Engineering for Matter, Transport and Energy, Arizona State University, Tempe, AZ 85281, USA; mark.sprowls@asu.edu (M.S.); svictor4@asu.edu (S.V.)
[2] Center for Bioelectronics and Biosensors, Biodesign Institute, Arizona State University, 1001 S McAllister Ave., Tempe, AZ 85281, USA; smora2@asu.edu (S.J.M.); oosoriop@asu.edu (O.O.); gpyznar@gmail.com (G.P.)
[3] Indoor Environment Group, Lawrence Berkeley National Laboratory, Berkeley, CA 94720, USA; hdestaillats@lbl.gov
[4] Mayo Clinic, Scottsdale, AZ 85289, USA; Wheatley.Courtney@mayo.edu (C.W.-G.); johnson.bruce@mayo.edu (B.J.); Kulick.Doina@mayo.edu (D.K.)
* Correspondence: eforzani@asu.edu

Citation: Sprowls, M.; Victor, S.; Mora, S.J.; Osorio, O.; Pyznar, G.; Destaillats, H.; Wheatley-Guy, C.; Johnson, B.; Kulick, D.; Forzani, E. A Smart System for the Contactless Measurement of Energy Expenditure. *Sensors* **2022**, *22*, 1355. https://doi.org/10.3390/s22041355

Academic Editor: James F. Rusling

Received: 23 December 2021
Accepted: 30 January 2022
Published: 10 February 2022

Publisher's Note: MDPI stays neutral with regard to jurisdictional claims in published maps and institutional affiliations.

Copyright: © 2022 by the authors. Submitted for possible open access publication under the terms and conditions of the Creative Commons Attribution (CC BY) license (https://creativecommons.org/licenses/by/4.0/).

S1. Smart Pad System Operating Equations and Measurement Technique

This supplementary information contains additional details on equations (1-7), including assumptions, the fundamental physical meaning of parameters, and reasoning for various aspects of analysis. The following equations were used during this study, all derived in previous work [25] as the basis for the EE measurements from the bulk environment of an occupied, enclosed setting (room, vehicle cabin, etc.). The following Equation only applies to the scenario where there are homogeneous CO_2 (all concentration units expressed in [ppm]) accumulating in the environment due to the presence of a human (a natural CO_2 production source), and there is some level of air exchange to the surrounding environment occurring. This effect (air exchange) is quantitatively characterized by λ [$hour^{-1}$], and specifically referred to as λ_{Acc} when it is applied to CO_2 accumulation data but with the same fundamental scientific meaning when applied to CO_2 decay data, where it s is referred to as λ_0:

$$[CO_2] = [CO_2]_0 + \frac{k_{gen}}{\lambda_{Acc}}\left(1 - e^{-\lambda_{Acc}t}\right) + \left([CO_2]_i - [CO_2]_0\right)e^{-\lambda_{Acc}t} \quad (1)$$

where $[CO_2]$ is the CO_2 concentration measured within the room (assumed to be perfectly mixed), $[CO_2]_0$ is the initial CO_2 concentration estimated via measurement of minimum CO_2 concentration with an occupant present and substracting 96 ppm from that number (a technique our team developed empirically via simultaneous measurement of chamber CO_2 and corresponding "source" CO_2 measurement at the same time point), $[CO_2]_i$ is the initial CO_2 concentration at the initial fitting timepoint, k_{gen} is the CO_2 generation rate [ppm $hour^{-1}$] due to the occupant's CO_2 production, and λ_{Acc} is the air exchange rate with the surrounding environment [$hour^{-1}$]. Its value mathematically represents the number of times the room's volume is

exchanged to the surrounding environment per hour $\frac{V_{Room}}{\dot{V}_{Room\,|\,Surroundings}}$ where V_{Room} is the volume of the room and $\dot{V}_{Room\,|\,Surroundings}$ is the volumetric flow rate of air exchanged between the room and its surroundings (room volume . hour^{-1}). Sample fitting of equation 1 is shown in the CO_2 accumulation portion of Figure 1C and many times throughout this supplementary materials document.

Previously [26], it had been determined that k_{gen} was correlated to the actual k_{gen}' via an environmental correction factor (CF_{env}) of 1.143, taken from a 20 subject study on the accuracy of the Smart Pad system, which is used in the model as follows:

$$k_{gen}' = k_{gen} CF_{env}. \tag{2}$$

Once λ_{Acc} is estimated via equation 7 (shown below), k_{gen} is then assessed for the subject and used to calculate k_{gen}' in Equation (2) above. k_{gen}' is then used in the following Equation to determine the value of VCO_2 [ml/min]:

$$VCO_2 = k_{gen}' * V_{Room} * CF_{STPD}/60 \tag{3}$$

where VCO_2 is the subject's volumetric production of CO_2 [ml/min], V_{Room} is the volume of the room [ml] (experimentally measured and accounting for the volume of objects within the environment), and CF_{STPD} [dimensionless] is a correction factor to correct the VCO_2 at ambient temperature and pressure conditions (ATP) to standard temperature, pressure, and dry conditions (STPD). The correction factor was calculated as follows:

$$CF_{STPD} = \frac{P_{bar} - P_{H20}}{760} * \frac{273}{T+273} \tag{4}$$

where P_{bar} [mmHg] is the barometric pressure inside the room measured with a barometric sensor, P_{H20} is the partial pressure of H_2O [mmHg] within the environment (with P_{Sat} calculated from the Antoine equation [69] and then also considering measured relative humidity (measured with a humidity sensor) % to find P_{H20}), and T is the temperature within the environment [Celsius] measured with a thermistor. The subject's EE [kcal/day] was calculated using a simplified version of the Weir formula [10] that assumes a constant respiratory quotient across subjects, or RQ, based on previously reported biomedical literature referenced below.

$$EE\left(\frac{kcal}{day}\right) = 3.941 * \frac{VCO_2}{RQ} + 1.106 * VCO_2 \tag{5}$$

$$RQ = \frac{VCO_2}{VO_2} \tag{6}$$

The RQ value used in equations 5-6 for REE calculation was 0.85, which, for a human following a mixed diet (relatively equal amounts of caloric intake from carbohydrate, fat, and protein) and meeting 100 ± 10% of caloric needs, is both theoretically [70] and empirically supported for subjects who are healthy [71,72]. Assumption of an RQ value is a generally accepted technique for measurement of EE, even for doubly-labeled water method (gold standard method) and FDA cleared REE measurement technologies

(e.g., Korr Reevue™: [67]). Figure S1 shows an example of CO2 data fitting with the equations mentioned above.

Finally, λ_0 was estimated from Equation (7):

$$[CO_2] = [CO_2]_0 + ([CO_2]_i - [CO_2]_0)e^{-\lambda_0 t}. \quad (7)$$

a standard first-order decay equation that is applied to CO_2 decay data occurring naturally after the subject exits the measurement environment. λ_0 was not previously correlated to λ_{Acc} from Equation (1) until this manuscript's publication.

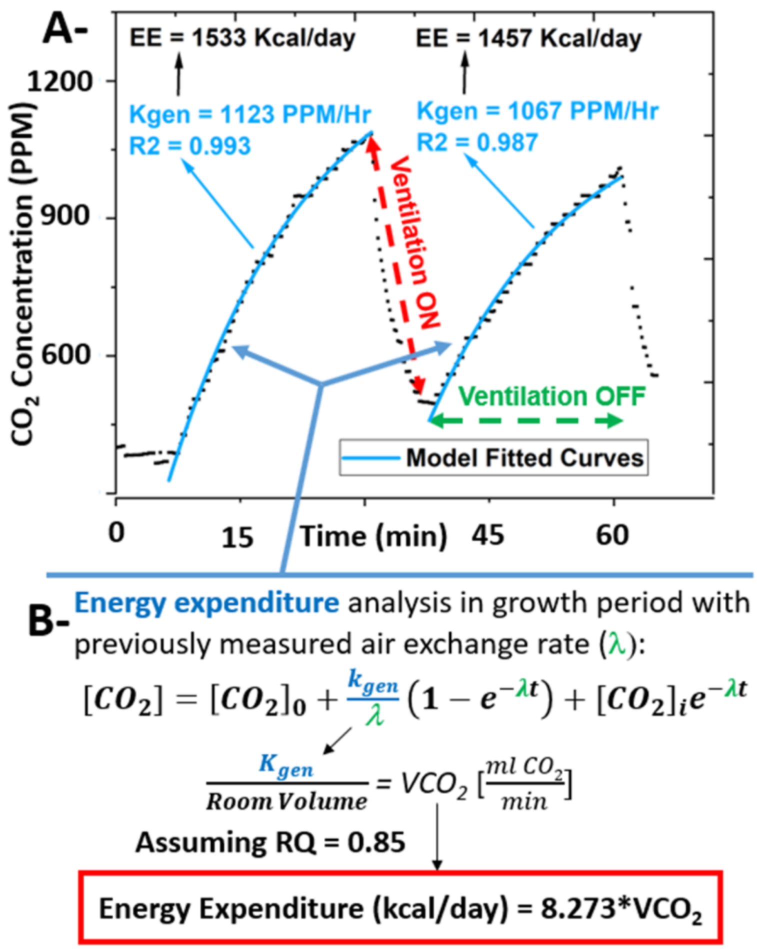

Figure S1. Panel A: Sample regressions for Smart Pad system with CO_2 concentration data analyzed in OriginLab™. Panel B: Simplified set of equations used for analysis

S2. Smart Pad: Physical Characteristics, Design, and Testing Environment

The Smart Pad measurement system's assembly is shown below in Figure S2. Further, Figure S3 shows how the Retrotec™ blower door was sealed for each experiment. For decay experiments, in particular, the subject would depart the room after CO_2 accumulation occurred to a predetermined level and would seal the Velcro on the Retrotec™ blower door. Figure S4 below shows how the actuator system was connected to

inlet and outlet fans. Finally, figure S5 below shows a control experiment performed to assure the carbon dioxide homogeneity assumption performed in the model (Equation 1).

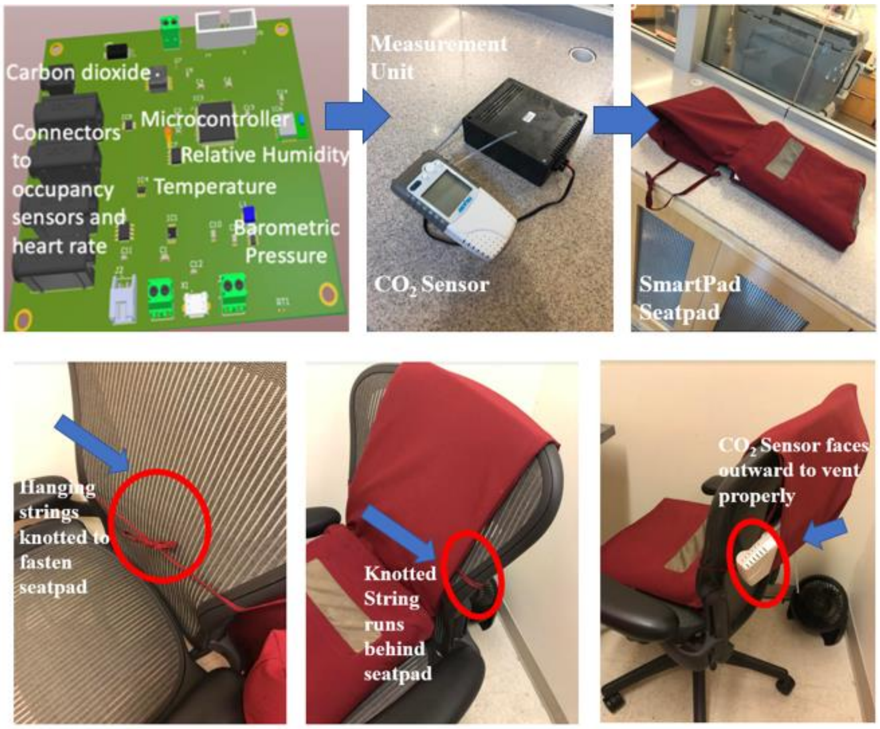

Figure S2. Graphic illustrating Smart Pad assembly.

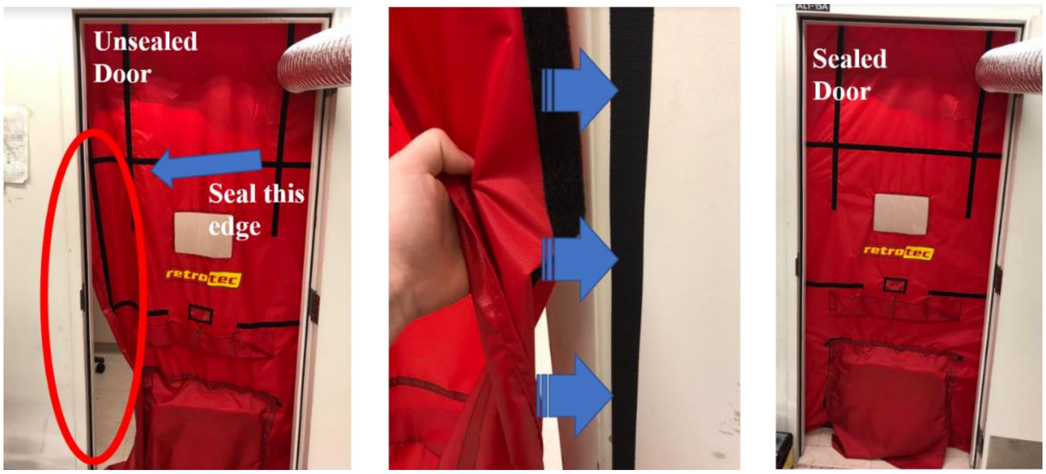

Figure S3. Graphic demonstrating how the Retrotec™ door was sealed for every experiment. Velcro lined on both the wall and the flexible door cover that allowed adequate room sealing. The aluminum duct in the upper right shows where the air was vented into the room (actively, when the Smart Pad app turned on the actuator system). Below the door's window, a fan covering can be seen. This opening was used to purge stale air out of the room via actuation of a 20in Lasko box fan (https://www.lasko.com/products/20-inch-box-fan-b20201/, accessed Feb. 2022).

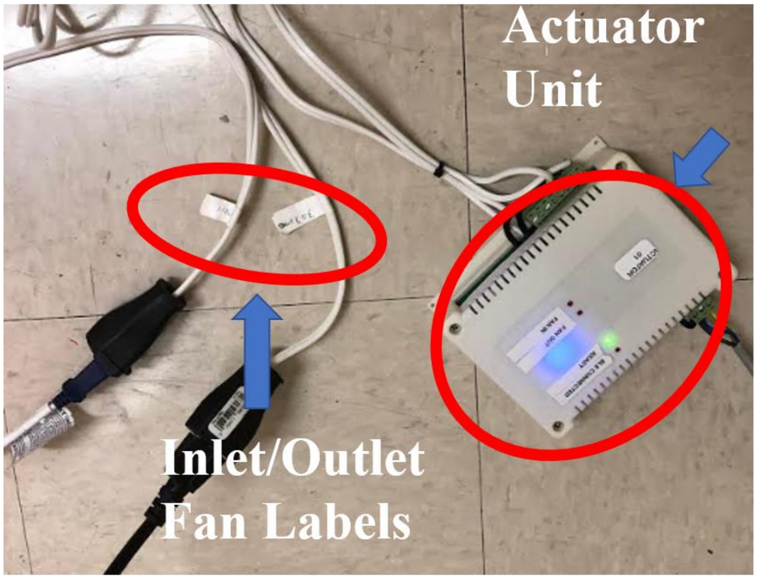

Figure S4. The Smart Pad actuator system.

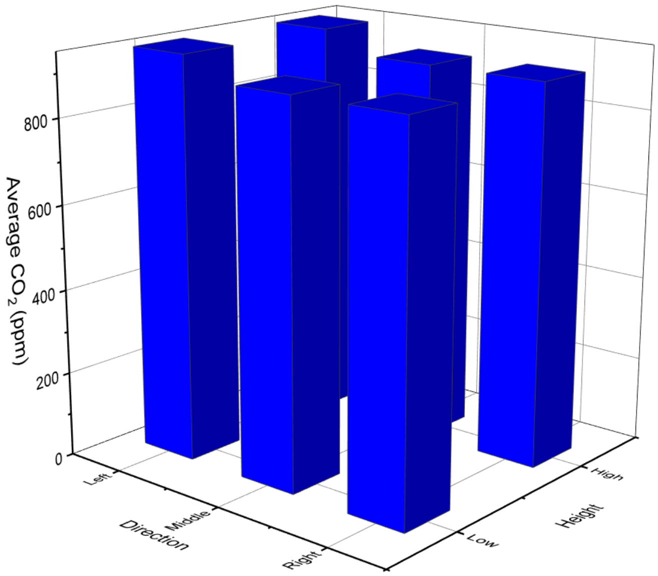

Figure S5. The Smart Pad room's carbon dioxide level distribution. The figure shows a control experiment where carbon dioxide levels were monitored at different heights and locations inside the Smart Pad room in the absence of a carbon dioxide source (i.e., $VCO_2 = 0$ mL/min) and with a minimum air exchange rate (i.e. λ = (0-0.5) h^1). The room was set as described in the experimental section, including two small fans. The constant levels of CO_2 throughout the room indicate that CO_2 was homogeneously distributed in the room and that the convection produced from the room's internal fans satisfied the carbon dioxide homogeneity assumption performed by the model (Eq. 1).

S3. CO_2 Accumulation Range Optimization for REE assessment

This section of the study was performed to build upon previous work published in the Journal of breath research [26], showing relatively good system accuracy in measuring VCO_2 from 20 minute CO_2 accumulation periods in a similarly sized room. This study strove to reduce the measurement time to 20 minutes without sacrificing measurement efficacy. To accomplish this, a single subject performed 113 REE measurements with both the Smart Pad and an FDA cleared [53, 68], reference instrument method for both VCO_2 and REE measurement, the MGC Ultima CPX™ (https://mgcdiagnostics.com/products/ultima-cpx-metabolic-stress-testing-system). Often 8-12 sequential CO_2 accumulation curves were collected on the same day, all analyzed using the λ_{Acc} value (measured using the reference instrument's VCO_2 reading) taken from the first CO_2 accumulation curve from that day. For each new day of collected data, a λ_{Acc} value was always extracted from the first CO_2 accumulation curve and applied to the CO_2 accumulation curves that followed that one. Since many CO_2 accumulation curves were used for λ_{Acc} assessment, there are a total of N=135 growth curve measurements, which allowed for an assessment of the effect of the CO_2 threshold range on time per measurement.

S4. CO_2 Accumulation Range Optimization for Exercise assessment

A stationary bike was located in the measurement environment (shown in Figure 2B), which was used to perform exercise EE measurements using the Smart Pad and reference instrument. Analysis was performed identically to the method of section 2.3, except that the λ_{Acc} assessment from reference instrument VCO_2 always assessed λ_{Acc} from a CO_2 accumulation curve occurring as a result of subject biking. The same set of threshold ranges was analyzed as in 2.3 (i.e., 500-600, 500-625, 500-650, 500-675, 500-700) for a total of N=40 measurements or N=8 measurements for each threshold range. Additionally, N=6 measurements were taken for an extended CO_2 threshold range of 500-900 ppm. VCO_2 measurement accuracy for the Smart Pad is the focus of this sub-study, and exercise EE is not reported. Reasons for this distinction are described in the main text and are primarily based on the difficulty of controlling subject RQ (a key parameter in EE measurement via the Weir formula as shown in equation 5) during the first 5 minutes of exercise.

S5. CO_2 Accumulation Range Optimization for REE assessment: Results

The authors suspect fitting in the reference instrument mask, and potential leaks in the facial accessory partly have influenced reproducibility (precision and final rated accuracy in this manuscript). Since fundamentally, the observed variance in the results is an additive product of the total errors in both Smart Pad and MGC Ultima CPX™ systems; one might expect the rated precision of the presented device to decrease if errors in reference instrument EE measurement were eliminated by using a more robust breath gas collection accessory (e.g., a mouthpiece accessory provided by the supplier).

S6. Additional information

Table S1. Physical Characteristics and Body Type Classification

Subject #	*Height (cm)*	*Weight (kg)*	*BMI (kg/m2)*	*Clinical Body Type Classification (CDC, 2021)*	*Age (Years)*	*Sex (male, female, or non-binary)*
1	178	79.4	25.1	Overweight	24	Male
2	153	44	18.8	Normal Weight	27	Female
3	185	107.3	31.4	Obese	26	Male
4	155	63.5	26.4	Overweight	35	Female
5	185	78.5	22.8	Normal Weight	25	Male

Sample data fittings for Subject #1:

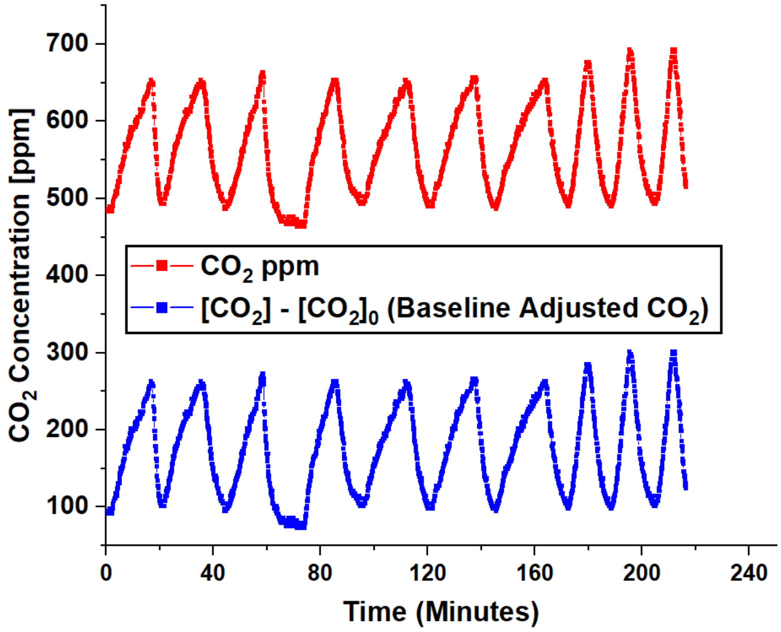

Figure S6. Sample raw data for many sequential REE assessments using a CO_2 threshold range of 500-650. The red curve shows the actual chamber CO_2 concentration measured from the Smart Pad. The blue curve shows the baseline adjusted CO_2 concentration, which is used to fit an identical model to Equation (1) but simplifies that the $[CO_2]_0$ term is subtracted from both sides of the Equation. Compared to no baseline adjustment, the tested approach of baseline adjustment gives an identical result both empirically on comparable CO_2 accumulation/decay data and mathematically by rearranging Equation (1) or Equation (7).

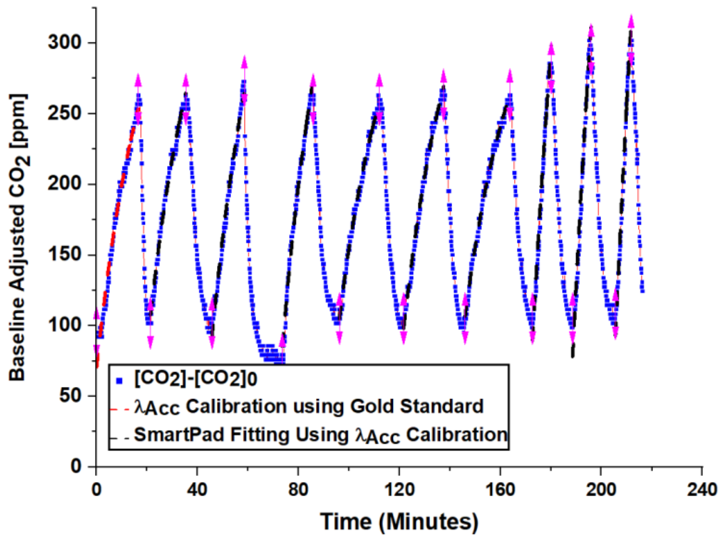

Figure S7. Sample baseline adjusted CO_2 fitting using Equation (1). The first CO_2 accumulation curve fits reference k_{gen} (calculated from reference instrument method VCO_2 measurement) to determine λ_{Acc}. Next, each sequential curve is analyzed with that same λ_{Acc} value. The last three CO_2 accumulation curves are for biking assessment (i.e., for section 2.4), the first being to calibrate for λ_{Acc} and the second and third for Smart Pad VCO_2/EE measurement.

Table S2. Figure S7 Analysis Results

Fitting # (Shown in Figure S7)	REE Smart Pad [kcal/day]	REE Reference instrument [kcal/day]	Error %
Accumulation fitting #1	2552	2609	-2.2
Accumulation fitting #2	2767	2618	5.7
Accumulation fitting #3	3012	2987	0.8
Accumulation fitting #4	2345	2187	7.3
Accumulation fitting #5	2504	2225	12.5
Accumulation fitting #6	2279	2199	3.7

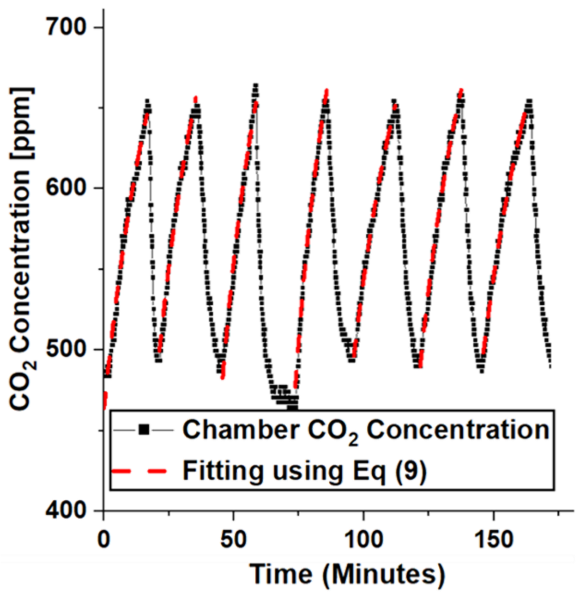

Figure S8. Sample fitting of equation 9 on CO_2 accumulation data. Data shown above were collected 2 months before the training set for equation 9, validating the new model's accuracy on a fully independent dataset.

Table S3. Figure S8 Analysis Results

**Accumulation Fitting # (Shown in Figure S8 as dashed black lines)**	**REE Smart Pad using Eq (9) [kcal/day]**	**REE Reference Instrument [kcal/day]**	**Error %**	**R^2 of Eq (9) Model**
Accumulation fitting #1	2242	2518	-11.0%	0.988
Accumulation fitting #2	2511	2609	-3.8%	0.990
Accumulation fitting #3	2856	2619	9.1%	0.986
Accumulation fitting #4	3295	2988	10.3%	0.990
Accumulation fitting #5	2144	2187	-2.0%	0.994
Accumulation fitting #6	2419	2225	8.7%	0.994
Accumulation fitting #7	2021	2199	-8.1%	0.994

S7. Discussion: Compensation for Imprecise Measurements using Repeated Measures

It is well known that repeated measurements can increase statistical confidence in a final result, even when a measurement instrument is relatively imprecise. This is a significant motivator and distinction between standard deviation and standard error. The standard error of a particular measurement is correlated with the inverse square root of the number of measurements. Assuming the same level of accuracy as was observed during this study (a large assumption, only posed to offer the reader the "vision" of the Smart Pad), a user of the device would potentially see a substantial increase in the confidence of their final average REE measure via repeated assessment (which could be relatively easy, given the Smart Pad performs contactless measurements). This could be in the form of multiple

monthly visits to a weight loss clinic or could potentially be in the form of 10 separate occupancy sessions (e.g., times that the subject has occupied the given environment for a minimum of 14-19 minutes) of a home study, bedroom, bathroom, vehicle cabin, or office space (e.g., inside a business/institution). For the Smart Pad measurement technique based on Equation (9), the realized accuracy across the N=56 measurements was 2.2±16.7% (68% CI).

Here, accuracy is defined as the maximum error expected for a given confidence interval based on experimental findings for the Smart Pad, as follows:

$$Accuracy\ \% = \ 100\% - (|Mean\ Error\ \%| + Standard\ Error) \quad (11)$$

Table S4. Figure S8 Analysis Results

Number of Repeated Measurements	Smart Pad Accuracy for Equation (9) (68% CI)
1	81.1%
3	88.2%
5	90.3%
10	92.5%

This extrapolation based on generally accepted consequences of standard error was further analyzed to account for the effect of total measurement time across multiple measurements. Here, the extrapolated results are for the 500-650 ppm CO_2 threshold range (where the 2.2±16.7% error was observed for the equation (9) model) was used, for which contactless REE measures were recorded in 14-19 minutes. For that reason, a mean measurement duration of 16.5 minutes was modelled below in Figure S9:

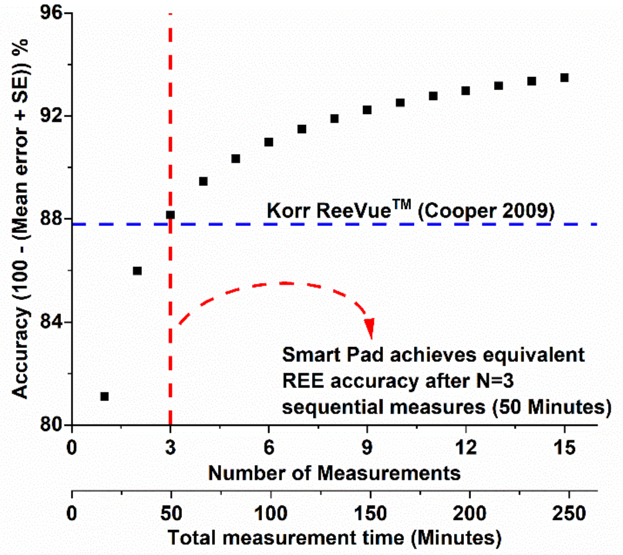

Figure S9. Smart Pad accuracy using Equation (9) and extrapolating based on widely accepted implications of standard error. Blue dashed line reflects the error reported by Cooper et.al. for "Korr Reevue™" instrument [21}.
